# Hypoplastic Left Heart: Stage-I Will be Performed Interventionally, Soon

**DOI:** 10.1007/s00246-021-02597-y

**Published:** 2021-04-19

**Authors:** Dietmar Schranz, Anoosh Esmaeili, Hakan Akintuerk

**Affiliations:** 1 Pediatric Heart Center, Justus-Liebig University, Feulgenstrasse 12, 35385 Giessen, Germany; 2 Department of Pediatric, Cardiology University Clinic, Frankfurt, Germany

**Keywords:** Hypoplastic left heart, Transcatheter, Palliation, Duct stenting, Flow restrictors

## Abstract

The hypoplasia of left-sided heart structures shows great variability and complexity. What the many variants have in common is that their heart structures are neither fully developed before nor after birth. Fetuses and newborns require an individual therapy depending on anatomy and function of the heart. Fetal interventions focus on improving left heart structures by catheter-based interventions and maternal hyperoxygenation which promotes growth as the left ventricular preload and blood flow within the cavity increase. Stage-I management of newborns with single ventricle physiology is usually based on the Norwood/Sano surgery or the Hybrid approach. Two more steps are required to ultimately achieve a Fontan circulation. Some centers also use the Hybrid approach for subsequent Norwood operation beyond the neonatal period. After the Hybrid approach, a comprehensive stage-II or corrective surgery is performed, the latter if a bi-ventricular circulation is possible. With progressively improved catheter-based interventions, particularly ductal stenting and manipulations of the atrial septum, the next advance is to develop a bespoke flow restrictor that can be easily inserted into the branches of the pulmonary artery. The main goal is to avoid complex heart operations under general anesthesia, followed by substantial intensive care in the neonatal period, especially for patients with complex heart defects. Based on the current state of the art of surgical treatment of hypoplastic left heart syndrome and variants with the Norwood surgery or the Hybrid approach, our main focus is on an alternative percutaneous transcatheter technique in the sense of a completely non-surgical stage-I approach.

## Introduction

**Fig. 1:**
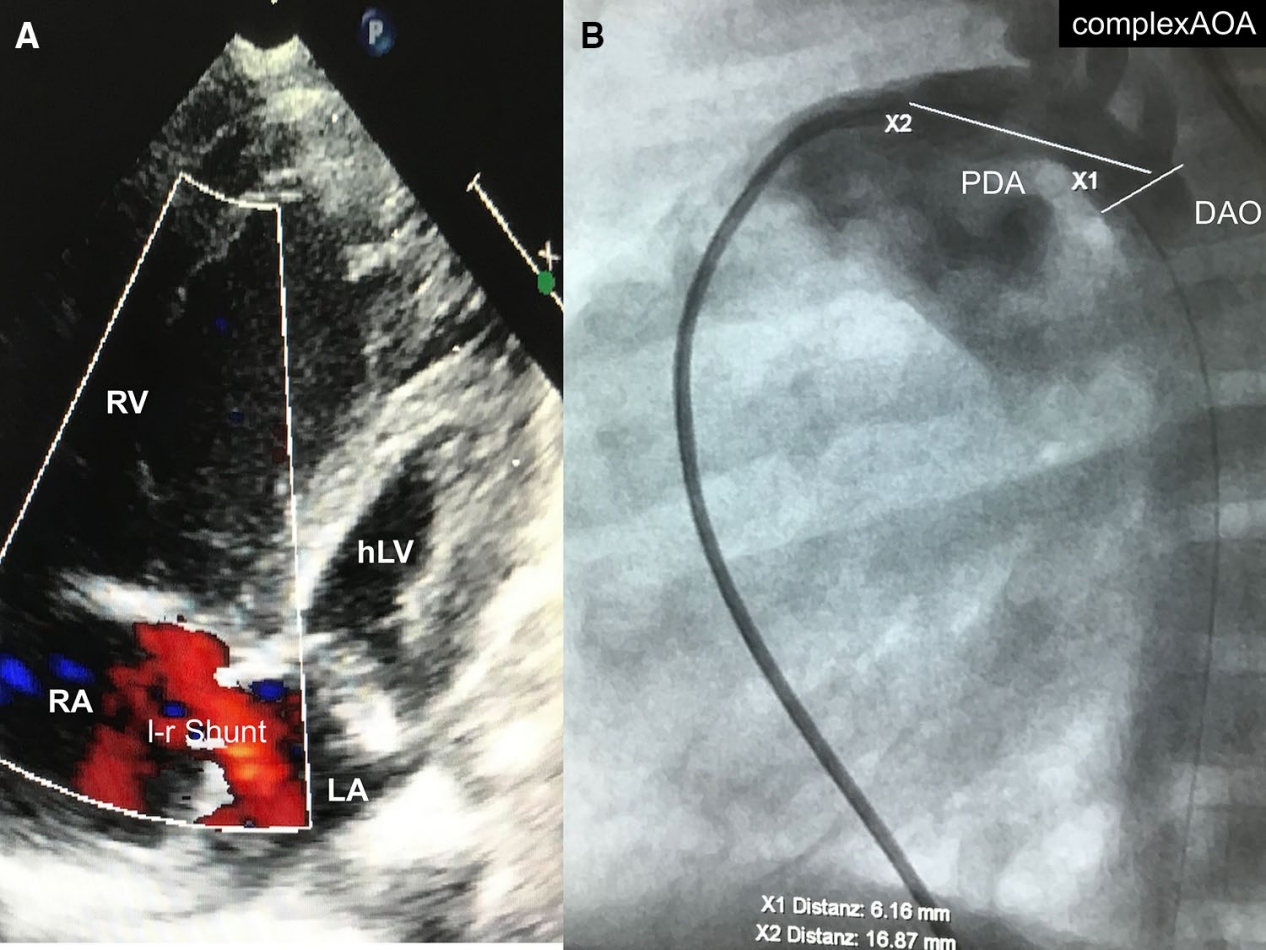
**a, b** Echocardiographic four-chamber view of a patient with HLHS (hypoplastic left heart syndrome) demonstrates left–right shunt across a non-restrictive atrial communication (**a**); lateral 90° angiography of right-left shunting, non-restrictive patent arterial duct (PDA) connected pulmonary artery to descending aorta (DAO) in a complex aortic arch (AoA) without clear differentiation of duct and aortic arch (**b)**. *hLV* hypoplastic left ventricle, *LA* left atrium, *RA* right atrium, *RV* right ventricle

The prevalence of congenital heart defects (CHD) is around 0.8–1% of all live births [1]. CHD accounts for almost 28% of deaths from birth defects in the first month of life and approximately 50% of the deaths during the first 12 months [2]. Hospital costs for pediatric CHD rose steadily and accounted for almost 23% of the total cost of all hospital stays for children and adolescents [2]. The majority of newborns with duct-dependent systemic blood flow are patients with hypoplastic left heart syndrome (HLHS), who make up 2–3.8% of CHD [1]. The hypoplastic left heart (HLH), however, encompasses a wide range of underdeveloped left-sided structures with stenosis or atresia of the aortic and / or mitral valve, variable sizes of the subaortic ventricle and variously shaped obstructions of the aorta. All anatomical variants have in common that the defects of the left side of the heart or of a hypoplastic right ventricle in connection with a congenitally corrected transposition of the great arteries (ccTGA) cannot pump enough blood to meet the requirements of the body. Therefore, the subpulmonary ventricle must ensure adequate systemic blood flow, which is supported by an atrial shunt from left-to-right and an arterial shunt from right to left and, if necessary, by ensuring adequate retrograde aortic flow (Fig. 1a, b).

Depending on the morphological conditions and their hemodynamic consequences, most newborns are still treated according to a three-stage surgical protocol. In rare cases, sophisticated bi-ventricular repair is performed during the neonatal period when morphological criteria allow such surgical decision-making [3].

This article describes the current state of percutaneous transcatheter techniques in order to gain an insight into the ideal basis for a complete, non-surgical stage-I palliation of newborns with HLHS and variants (Table 1).

**Table 1:**
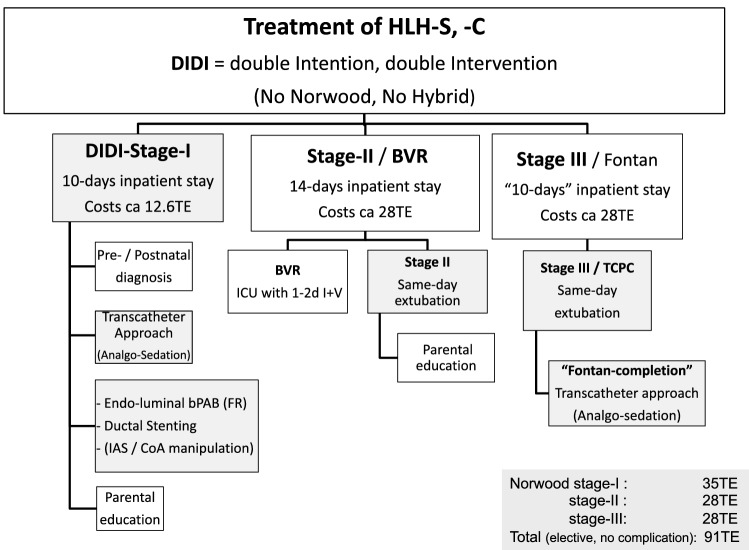
Intended treatment of HLHS, HLHC costs, institutional-based calculated

bPAB bilateral pulmonary arterial banding, BVR bi-ventricular repair, CoA coarctation, IAS interatrial septum, ICU intensive care unit, TE Thousands of Euro

## Traditional Norwood Stage-I Palliation and Follow-up Results

Norwood stage-I consists of advanced neonatal surgery with cardiopulmonary bypass (CPB) sometimes with circulatory arrest in deep hypothermia or selective cerebral perfusion. The aortic arch is reconstructed within the first days of life in combination with a Blalock-Taussig or, alternatively, a (right) ventricular pulmonary artery (Sano) shunt [4, 5]. Stage-II is usually required in a 3 to 6 month follow-up with the superior vena cava connected to the pulmonary branch (bidirectional Glenn anastomosis), followed by stage-III (Fontan surgery), which is typically performed within the first 2 to 4 years [6, 7]. The alternative technique known today as the Hybrid approach was first carried out by Gibbs et al. [8]. However, due to the failed hybrid procedure in the first eight newborns, the Leeds group did not recommend the procedure [9], although a successful collaborative surgical-interventional approach had already been implemented in Giessen [10]. The Giessen-Hybrid approach (GHA) consists of a surgically performed bilateral pulmonary artery banding (bPAB), followed by percutaneous duct stenting and, if necessary, atrial septum or aortic isthmus manipulation. Comprehensive stage-II, which is usually performed at 4 months of age, was first successfully performed by Hakan Akintuerk in 1998 [11]. GHA switched from a rescue procedure to an elective approach, replacing the Norwood procedure as initial palliation for newborns with HLHS [12]. The Hybrid approach is also the strategy of choice for patients with borderline left heart structures to avoid high-risk bi-ventricular repair as a newborn and to lay the basis for further postnatal growth of the subaortic chamber [13, 14]. At the beginning of the new millennium, another collaborative surgical-interventional group in Columbus, Ohio focused on a one-step Hybrid procedure, in which the arterial duct is stented using a transpulmonary access immediately after the bPAB is placed [15]. In Sao Paulo, Brazil the Hybrid variant has now also replaced the Norwood procedure [16]. Several centers around the world are currently using the Hybrid approach for newborns with HLHS or HLH complex after high-risk assessments to perform delayed Norwood surgery or bi-ventricular repair and for bridging to heart transplantation [17–23]. If a patient’s ventricle is too small for postnatal BVR, the hypoplastic ventricle must be rehabilitated using a balanced “surgical-interventional” procedure that allows adequate blood flow in the ventricular cavity for further postnatal growth of the borderline left heart structures [24]. The requirements for the Norwood procedure have not changed. There is still a need for (1) unimpeded systemic blood flow (SBF) generated by a single “right” ventricle or together with a hypoplastic subaortic ventricle to a reconstructed ascending aorta and aortic arch (2) a controlled pulmonary blood flow (PBF) based on an unimpeded pulmonary venous return. PBF is ensured by a modified Blalock-Tausig shunt (mBTS) or by a (right) ventricle-to-pulmonary artery shunt (RVPAS). The RVPAS reduces diastolic run-off with subsequent coronary arterial steal phenomenon, which is common in mBTS, but at expense of a ventriculotomy; a potential risk of single ventricular dysfunction and arrhythmia generation [25]. Based on the immense data of Pediatric Heart Network [26, 27], adequate 12-month follow-up data with multiple endpoints such as mortality or heart transplantation (HTx) are available. It includes stage-II surgical results, medium-term dominant function of the right ventricle, the fate of the pulmonary artery, and results of neurodevelopmental. Several risk factors for death were analyzed. With a mean follow-up of almost 3 years, there was only one significant benefit of RVPAS in patients with an atretic aortic valve [14]. The total mortality or the need for HTx- was 33% in patients with RVPAS versus 39% in mBTS [27]. Despite the highest mortality of all congenital abnormalities, the management of HLHS by the Norwood method is resource-intensive [28]. Institutional experiences showed that stage-I hospitalization with an average length of stay of 34 (24–58) days resulted in average cost was $203,817 (136,000–272,000). The average survivors’ costs at 12 months were $369,393 ($216,000–594,000). The main costs related to the intensive care unit (41%), the hospital outside the ICU (17%), the surgical service (11%), catheterization laboratory (9%), and the pharmacy (9%). The cost of HLHS management continues to be determined by complications such as reoperation, the need of extracorporeal membrane oxygenation (ECMO), and patient factors such as low birth-weight [28]. Recently published multicenter data [29] showed an analysis of 2872 Norwood records in HLHS over a five-year period between 2003 and 2016. Mortality decreased from 28 to 14%, although the incidence of ECMO increased from 10% to 17.3%. ECMO-related mortality was 55% vs 11% of non-ECMO patients, but ECMO had a significant impact on cost. Unlike the USA, which tends to shorten hospital stays, some European pediatric heart centers prefer hospitalization to improve interstage mortality following Norwood surgery, but without a cost analysis similar to that in the USA [30].

## Current Hybrid Procedures and Follow-up Results

The goal of the Hybrid approach to treating newborns with HLHS is identical to the Norwood procedure. However, advanced cardiopulmonary bypass surgery within the endangered neonatal period is avoided [10, 31]. The Hybrid surgical-interventional approach must achieve adequate systemic blood flow through an unobstructed arterial duct and a free aortic arch through ductal stenting or continuous long-term prostaglandin E1 infusion [32–34]. The lungs are protected by attaching left and right pulmonary artery branch banding [31, 32] in order to achieve a balanced pulmonary and systemic circulation, provided that the atrial communication is not obstructed. Since the use of PTFE strips [32], the procedure has been simplified and some historical problems with the potential for loss of the left superior pulmonary artery segment during de-banding have been nearly resolved. However, most surgeons prophylactically patch the LPA or insert a stent during the comprehensive stage-II procedure to avoid postoperative problems with the LPA [32]. Right from the start, the GHA concept was based from the beginning on a sequential procedure starting with bilateral PAB as an open-chest surgery followed by elective duct stenting and needs-based manipulation of the atrial septum or the aortic isthmus as a percutaneous transcatheter measure [10, 11, 35]. Percutaneous stenting of the arterial duct is usually performed in sedated newborns, and possibly atrioseptostomy or manipulation of the aortic isthmus, if necessary [33–35]. Another advantage of the percutaneous duct stenting that goes beyond safety is the ability to analyze the isthmus area of the aorta before and after stent placement [37]. Aortic isthmus obstruction can be effectively treated by using an additional coronary stent or a specially designed self-expanding stent [11, 35]. If technical and material details are taken into account, percutaneous duct stenting can be performed with almost no mortality [11, 12]. The detailed technique of duct stenting, and manipulation of atrial septum and aortic coarctation were recently described as part of the AEPC/EATS HLHS guidelines [36]. The interstage remains a vulnerable period, that requires closed monitoring of the infants. In addition, we developed a drug regimen consisting of long-acting selective ß1-blocker (B, bisoprolol) for almost all patients in combination with tissue ACE-inhibitor (L, lisinopril) and mineralocorticoid blocker (S, spironolactone), provided there are obvious contraindications are excluded. The aim is to protect the myocardium of a single ventricle and to economize the cardiovascular system, should be supported together with a low systemic vascular resistance without endangering the coronary and cerebral perfusion pressure. A moderately restrictive atrial septum defect with a pressure gradient of 5 to 15 mmHg targets a borderline LV physiology that favors blood flow within the ventricular cavity; the indispensable prerequisite for the growth of the subaortic hypoplastic ventricle with its potentials for bi-ventricular repair in later infancy [13, 24]. In today’s experience, when the infant reaches a body weight of 5 to 6 kg by the age of 4 to 5 months, bPAB with a diameter of 3.5 mm provides adequate protection for the lung bed without becoming too tight. Retrospective data from centers that have replaced Norwood operation with the Hybrid approach as a first-line procedure show that comprehensive stage-II is performed with less than 5% mortality [12, 37, 38]. The advanced surgical approach consists of removal of bPAB and ductal stent, full atrial septectomy, bidirectional cavo-pulmonary connection (BCPC) with patch augmentation, or stenting of the left pulmonary artery, followed by a Norwood-style reconstruction of the aortic arch with removal of the isthmus area and the entire ductal tissue with stent material. The financial impact of a complication-free Hybrid approach in our facility in Germany, in which the costs for stage-I and -II are combined, amount to 63,000 Euros (TE), with a maximum reimbursement of 87TE. Stage-I Norwood surgery is paid with 35TE (maximal 50TE, if ventilation time exceeds 125hrs) followed by stage II for approximately 28TE (maximum 37 TE, if ventilation time exceeds 95hours). The Hybrid procedure with stage I and the subsequent comprehensive stage II is calculated together, but is overall identical. Even so, the cost of Hybrid stage-I is significantly lower than that of Norwood stage-I, but the comprehensive stage II is more expensive, than that of Norwood stage-II. The need for ECMO is paid for additionally. In the last decade, five out of exactly 100 patients had a need for ECMO after comprehensive stage-II. This results in an overall ECMO incidence of 5%; the mortality rate was 2%. Both patients were also treated with ECMO. The German health care system covers currently all patient’s costs.

### Future Approach for Palliation of HLH (S) by Transcatheter Techniques

**Fig. 2:**
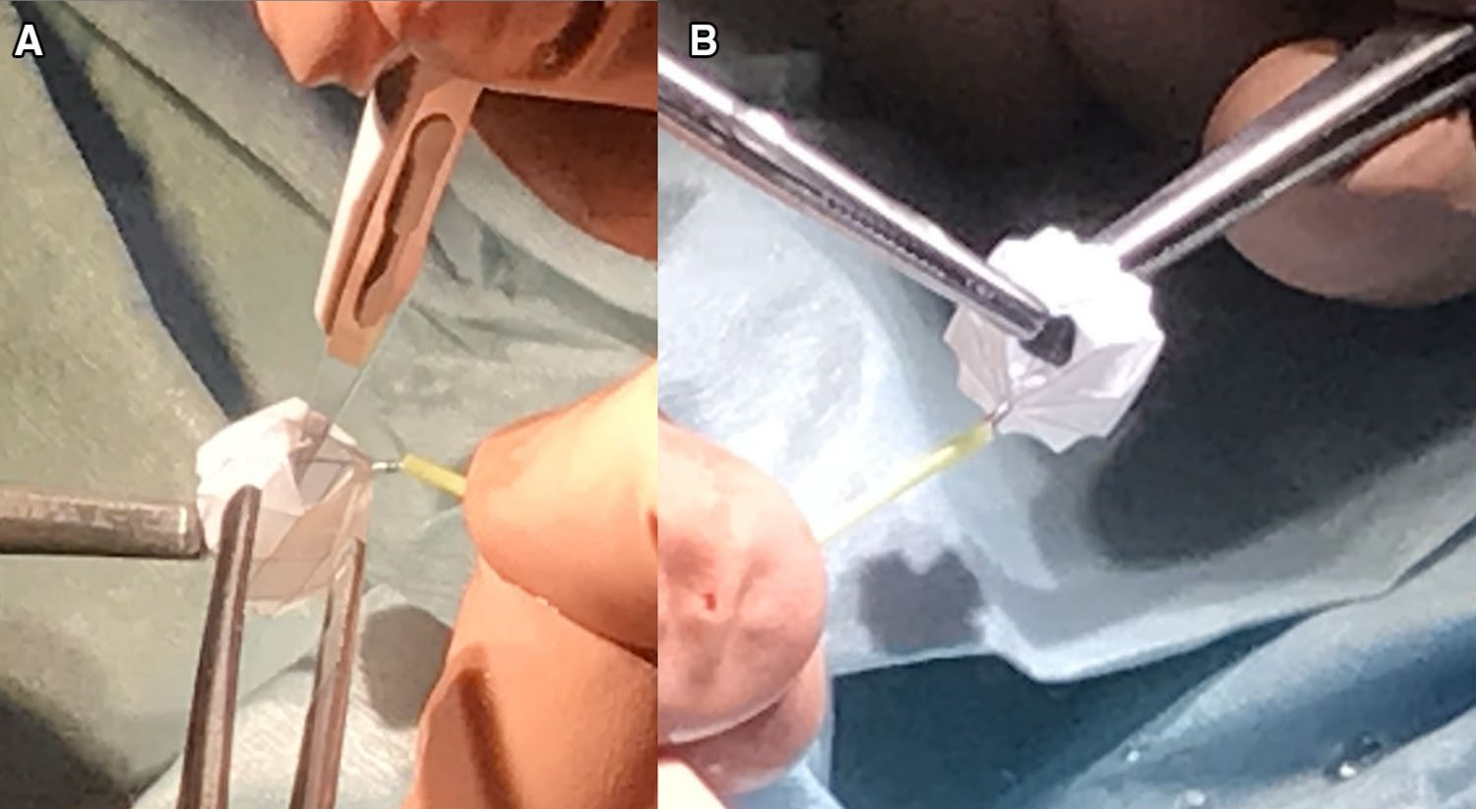
a, b Shown is a manually modified, CE-marked Medtronic® Micro Vascular Plug ™ (MVP-Q7) as a pulmonary flow restrictor (PFR) by removal of PTFE® from one end cell by scalpel (**a**) and the final result (**b**)

The future has already begun [39]. The first six newborns with HLHS and variants also survived the follow-up operations with remarkable good results; however, one patient suffers from a syndrome-related liver dysfunction after HTx. Four patients have successfully completed comprehensive stage-II and one infant an uneventful bi-ventricular repair [39]. Based on sophisticated animal studies [40], pulmonary artery flow restrictors (PFRs) were manually created using CE-marked Micro Vascular Plug devices (MVP-5Q, 7Q or 9Q, Medtronic™) with sizes originally intended to accommodate vessels up to 5, 7 or 9 mm diameter (Fig. 2a, b). The novel transcatheter procedure was administered as compassionate treatment with the written parental consent and in accordance with our institutional ethical guidelines [39]. Meanwhile, three other patients with fetal and neonatal left ventricular obstructions received an uneventful transcatheter stage-1 approach, awaiting a follow-up bi-ventricular repair in two cases and a comprehensive stage-II soon. A prospective multicenter study is already underway.

## Technical Aspects of Percutaneous Stage-I Approach

**Fig. 3:**
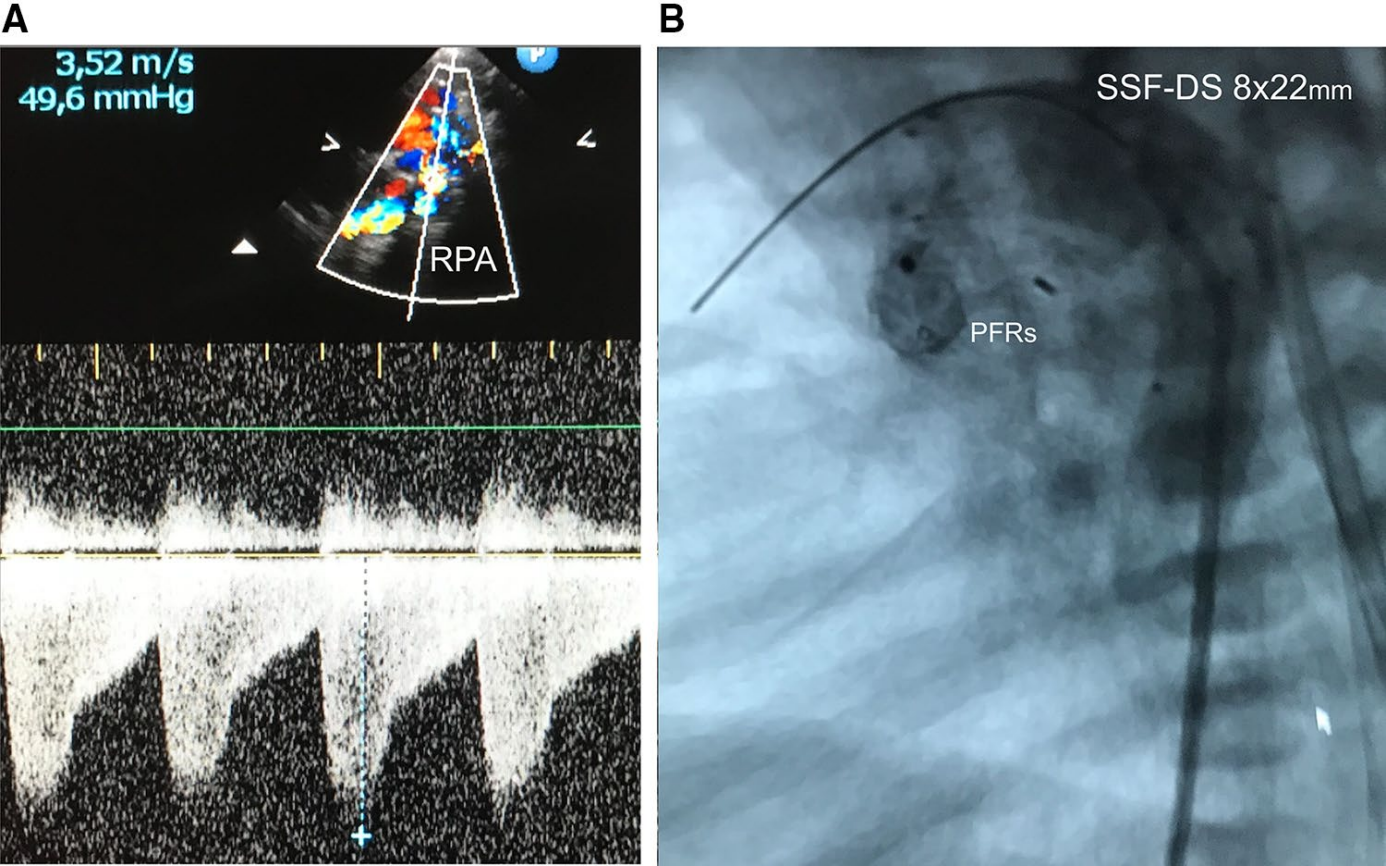
**a**, **b** Two-D- and color-Doppler echocardiographic short axis plane depicts the right pulmonary artery (RPA) and the belonging continuous wave (CW) Doppler tracing pulmonary systolic-diastolic flow profile representing an effective PFR (pulmonary flow restrictor, **a** lateral 90° angiography shows the final right-to-left shunt across the stented arterial duct using a SinusSuperFlex-DS stent (SSF-DS) and PFRs within the pulmonary artery branches **b)**

**Fig. 4:**
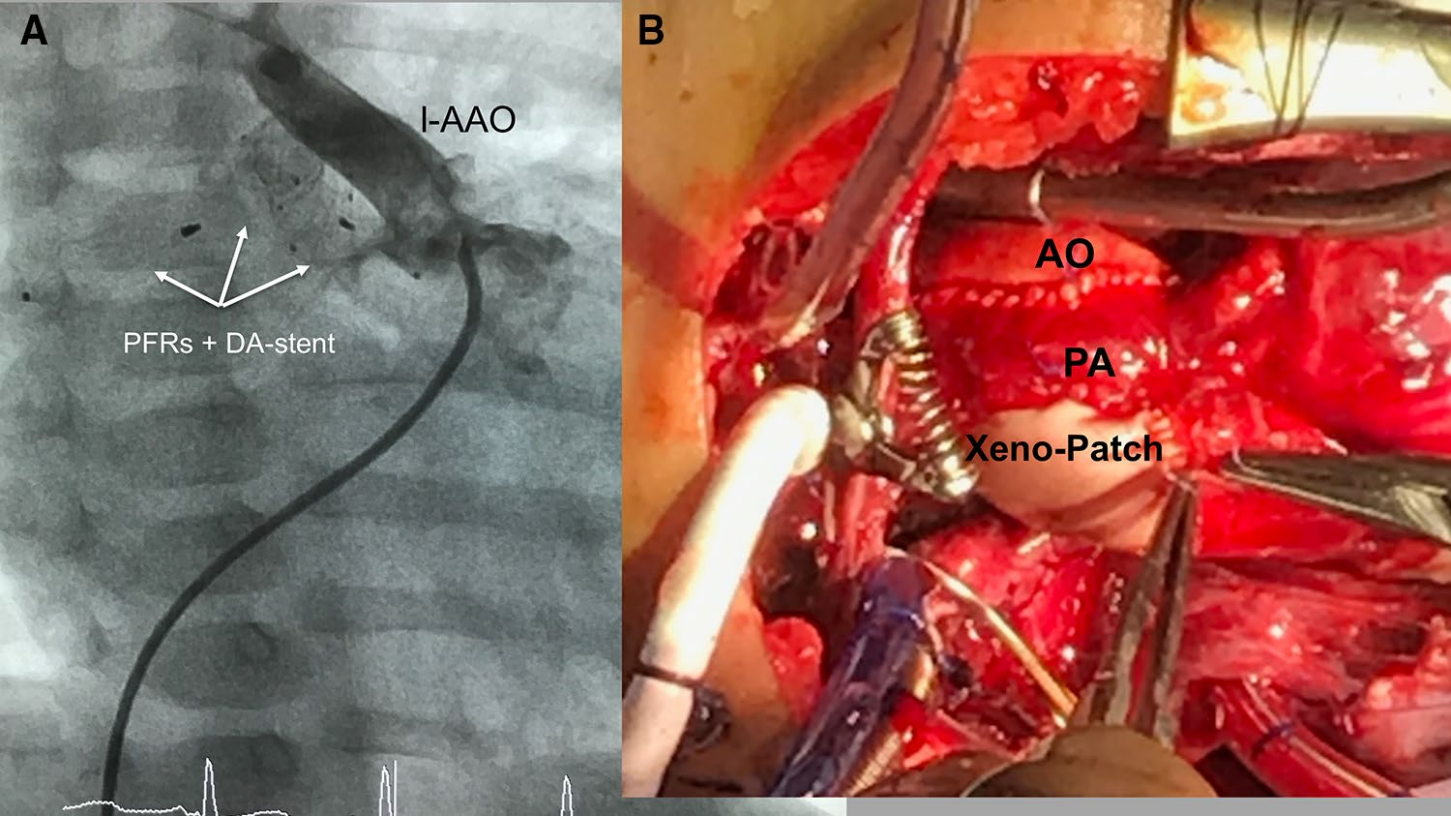
**a**, **b** Angiography of a patient with congenital corrected transposition (ccTGA) with left anterior positioned ascending aorta (l-AAO) palliated with transcatheter performed stage-I by PFR (pulmonary flow restrictors) and ductal stenting (DA) prior to the comprehensive stage-II (**a**); figure **b** shows the final result of the reconstructed ascending aorta, necessarily different in a ccTGA patient to a usual performed “Norwood-like” repair. Pulmonary artery (PA) is right side connected to the ascending aorta (AO) and the curvature of the aortic arch with xeno-pericardium®

**Fig. 5:**
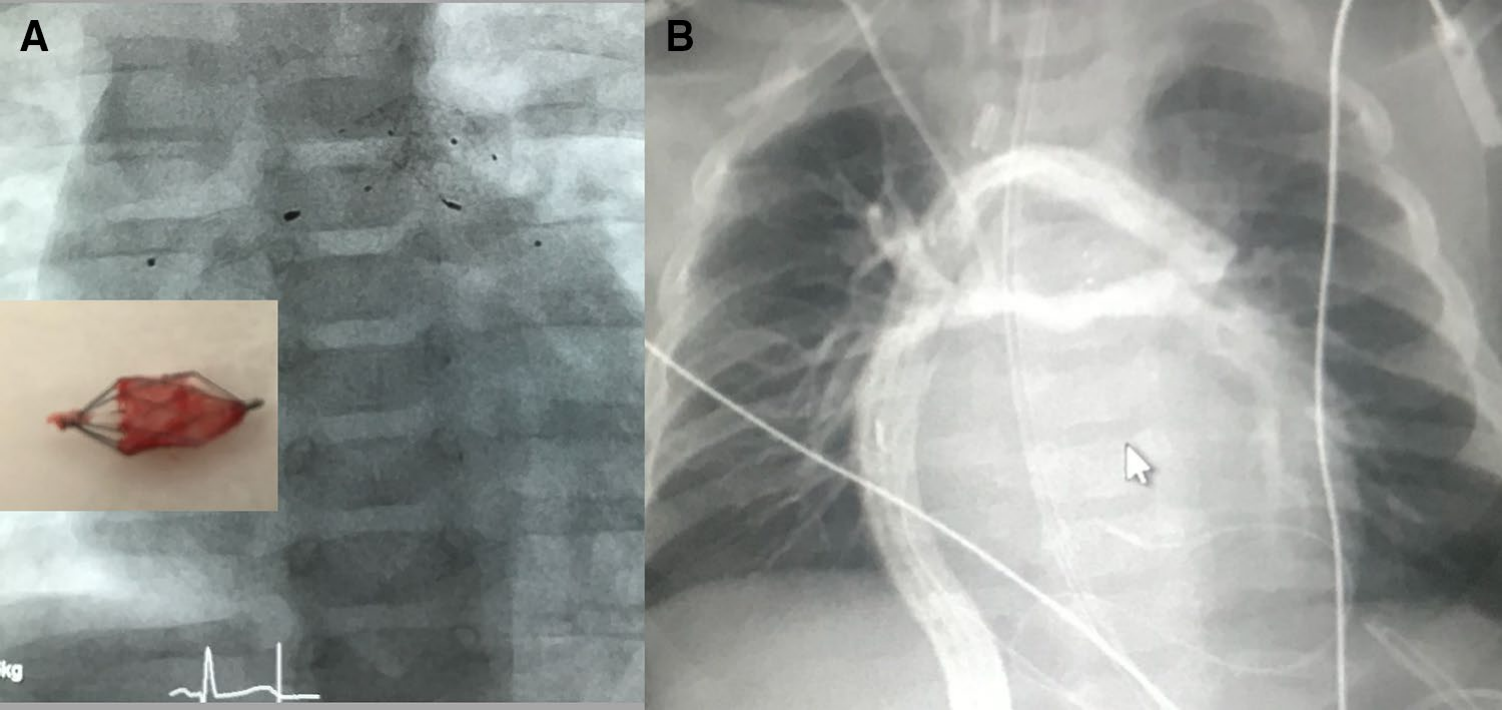
**a**, **b** Anterior–posterior x-ray of a ccTGA patient treated with PFRs and ductal stent; shown is also a removed MVP-based flow restrictor (**a**); figure **b** shows the contrast-agent-filled pulmonary artery system immediately after comprehensive stage-II surgery

With regard to the “dual” intention of avoiding Norwood surgery or the surgical part of a Hybrid approach through a “double” intervention consisting of the placement of endoluminal pulmonary flow restrictors (PFR) and subsequent duct stenting, percutaneous transcatheter procedure is summarized as “DIDI”-approach. The objectives of the “DIDI”-approach are the same as those of the Hybrid procedure. Regardless of whether a fetal diagnosis has already been made or whether the newborn was born with an undiagnosed congenital heart defect and already shows symptoms of congestive heart failure, the “DIDI” -approach can be used, also with complex morphologies (Figs. 3, 4). Stage-I transcatheter palliation is also feasible without direct surgical facilities for congenital heart defects and regardless of the socio-economic status. Percutaneous cardiac catheterization is usually performed on a spontaneously breathing, sedated newborn through access to the femoral vein. Depending on the technique, for the intervention only a 4Fr sheath is required in the femoral vein. Using a 4Fr Right-Judkins (RJ) or 4Fr Cobra-shaped (Terumo®) diagnostic catheter placed at the junction of the inferior caval vein and the right atrium, a soft-tipped coronary wire is passed through the right ventricle, pulmonary trunk and ductus arteriosus (DA) within the descending aorta (DAO) followed by the RJ or Cobra catheter. While the catheter is being withdrawn from the DAO over the still positioned coronary wire, the DA morphology is delineated by injecting contrast medium per hand. The angiographically obtained dimensions of the DA form the basis for the selection of a suitable stent. In the case of an unhindered DA caused by continuous PGE1 infusion of 5 to10ng/kg/min, a self-expandable Sinus-Superflex stent (SSF-DS Optimed®, Karlsruhe) with a diameter of 1-2 mm larger than the smallest diameter of the duct is used, but definitively larger than the DAO diameter. In the event of an obstructed duct, a balloon expandable stent is preferred, but it requires a 5 or even 6Fr sheath within the femoral vein. In general, duct stenting is performed after the PFRs are inserted into the branches of the pulmonary artery. Therefore, the diagnostic catheter is withdrawn within the PA trunk and the coronary wire pulled back from the DAO and directed into the LPA first. After selective angiography to assess the diameter and length until the distal branch of the LPA, a modified MVP-5Q can be implanted using the same RJ/Cobra catheter. MVP-7Q- or MVP-9Q-based PFR’s require a 0.038inch lumen catheter as the Terumo® 4Fr Cobra glide catheter. Given that the right pulmonary artery (RPA) is normally larger than the LPA in a newborn with HLHS, a modified MVP-7Q (9Q) is usually used, sometimes also a modified MVP-9Q. It must be emphasized that the MVP-based PFR is placed into a high-pressure vascular circuit. This means that the ratio of the device to the measured vessel lumen should be chosen generously to a larger PFR size, as is normally recommended. The effectiveness of the PFR can be demonstrated by echocardiography. A systolic-diastolic Doppler flow pattern represents an effective PFR (Fig. 3a). Therefore, an invasive pressure measurement with a pressure-wire or catheter retraction technique through the PFR in LPA and RPA is not required. Based on our experience with the surgical PAB-technique while performing the Giessen-Hybrid approach, effective PFRs are achieved by removing PTFE from every seventh (MVP-5Q) or one of ten PTFE covered end-cells (MVP-7Q, 9Q) of the self-expanding device. This corresponds with holes of about 3 to 4 mm. Due to the institutional experience, the following placement of a duct stent is technically an uncomplicated approach (Fig. 3b). If necessary, manipulation of the atrial septum must also be performed. However, the overall success of the “DIDI”- transcatheter approach seems to be more related to close follow-up monitoring during the remaining vulnerable interstage. Usually, clopidogrel is administered in one oral dose of 0.2 mg/kg/day [41], rarely combined with a low-dose (1-2 mg/kg/day) of acetylsalicylic acid. We argue that the shape of the self-expanded MVP with manual change to a PFR by removing the very thin PTFE membrane from only one end-cell and the flow properties achieved with it, the risk of thrombus formation, but also hemolysis, is low. Bisoprolol is administered to each patient in a once daily dose of 0.1–0.2 mg/kg. Lisinopril and spironolactone can optionally also be used once a day, which is limited according to the Hybrid approach. An operating date for a comprehensive stage-II is already set 4 months after the “DIDI” approach. Different to the comprehensive stage II after the Hybrid approach with removal of the bilateral PAB, bilateral PFRs are removed using a dissector technique. Following the bidirectional Glenn anastomosis, the aortic arch is reconstructed in a similar way to the Norwood repair without deep hypothermia, but must be adapted to the individual anatomy (Fig. 4a, b). A patch expansion of the LPA or prophylactic stent placement should not be necessary, which facilitates and shortens the comprehensive stage-II surgery. Considering that the endothelium is temporarily affected by removing the devices in a four-month follow-up (Fig. 5a), the heparin antagonization is carried out restrictively, sometimes the patient is admitted to the intensive care unit. The pulmonary arteries are assessed by an exit angiography or by the first thoracic x-ray after arrival at the intensive care unit by applying contrast medium per hand through the subclavian or jugular venous catheter (Fig. 5b). Extubation takes place a few hours after the operation. The patient is discharged home, when the oxygen-free arterial oxygen saturation ranges between 75 and 85% and after maternal re-education to care for her baby.

## Conclusion

A hybrid approach can replace the sophisticated neonatal Norwood procedure [14]. Today it can be hypothesized that the initial postnatal stage-I can also be performed without surgery in almost all newborns with complex congenital heart defects with duct-dependent systemic circulation. Transcatheter interventions can be carried out as elective therapy, but also under conditions of high urgency. In the view of the technical development of duct stenting and in particular the percutaneous application of endoluminal PAB, not only the goal of avoiding neonatal Norwood operation comes closer, but also the part of the Hybrid approach as a surgical intervention on the open chest for the placement of bilateral PABs in newborns with HLHS and HLHC. Based on a bespoke PFR that can be launched via a “mini” supply catheter, we are confident that great strides can soon be made in completing the stage-I using complete transcatheter technique as standard basic care. A prospective, multicenter study is warranted.
